# Is Better Standardization of Therapeutic Antibody Quality in Emerging Diseases Epidemics Possible?

**DOI:** 10.3389/fimmu.2022.816159

**Published:** 2022-02-22

**Authors:** Sanda Ravlić, Ana Hećimović, Tihana Kurtović, Jelena Ivančić Jelečki, Dubravko Forčić, Anamarija Slović, Ivan Christian Kurolt, Željka Mačak Šafranko, Tatjana Mušlin, Dina Rnjak, Ozren Jakšić, Ena Sorić, Gorana Džepina, Oktavija Đaković Rode, Kristina Kujavec Šljivac, Tomislav Vuk, Irena Jukić, Alemka Markotić, Beata Halassy

**Affiliations:** ^1^ Centre for Research and Knowledge Transfer in Biotechnology, University of Zagreb, Zagreb, Croatia; ^2^ Center of Excellence for Virus Immunology and Vaccines (CERVirVac), Zagreb, Croatia; ^3^ Croatian Institute of Transfusion Medicine, Zagreb, Croatia; ^4^ Research Department, University Hospital for Infectious Diseases “Dr. Fran Mihaljević”, Zagreb, Croatia; ^5^ Clinics for Pulmonary Diseases, University Hospital Centre Zagreb, Zagreb, Croatia; ^6^ Department of Hematology, University Hospital Dubrava, Zagreb, Croatia; ^7^ Department for Transfusion Medicine, University Hospital Dubrava, Zagreb, Croatia; ^8^ Department for Clinical Microbiology, University Hospital for Infectious Diseases “Dr. Fran Mihaljević”, Zagreb, Croatia; ^9^ School of Dental Medicine, University of Zagreb, Zagreb, Croatia; ^10^ Clinical Institute for Transfusion Medicine, Clinical University Hospital Centre Osijek, Osijek, Croatia; ^11^ School of Medicine, Catholic University of Croatia, Zagreb, Croatia; ^12^ Faculty of Medicine, University of Rijeka, Rijeka, Croatia

**Keywords:** passive antibody therapy, convalescent plasma, COVID-19, SARS-CoV-2, wild-type virus neutralization assay

## Abstract

During the ongoing COVID-19 epidemic many efforts have gone into the investigation of the SARS-CoV-2–specific antibodies as possible therapeutics. Currently, conclusions cannot be drawn due to the lack of standardization in antibody assessments. Here we describe an approach of establishing antibody characterisation in emergent times which would, if followed, enable comparison of results from different studies. The key component is a reliable and reproducible assay of wild-type SARS-CoV-2 neutralisation based on a banking system of its biological components - a challenge virus, cells and an anti-SARS-CoV-2 antibody in-house standard, calibrated to the First WHO International Standard immediately upon its availability. Consequently, all collected serological data were retrospectively expressed in an internationally comparable way. The neutralising antibodies (NAbs) among convalescents ranged from 4 to 2869 IU mL^-1^ in a significant positive correlation to the disease severity. Their decline in convalescents was on average 1.4-fold in a one-month period. Heat-inactivation resulted in 2.3-fold decrease of NAb titres in comparison to the native sera, implying significant complement activating properties of SARS-CoV-2 specific antibodies. The monitoring of NAb titres in the sera of immunocompromised COVID-19 patients that lacked their own antibodies evidenced the successful transfusion of antibodies by the COVID-19 convalescent plasma units with NAb titres of 35 IU mL^-1^ or higher.

## Introduction

Passive immunotherapy is a century-old practice of administering pathogen-specific antibodies to prevent or treat a disease caused by the same pathogen (1). Specific immunoglobulins (pooled, purified and concentrated immunoglobulin preparations), some even sourced from animals, have an important role in the prophylactic or therapeutic treatment of various clinical conditions, including viral diseases (hepatitis A and B, rabies, varicella, infections with respiratory syncytial virus, cytomegalovirus, measles). However, in situations with insufficient time or resources to generate the immunoglobulin preparations, such as during emerging infections and pandemics (influenza, SARS-CoV-1, MERS, Ebola), convalescent plasma can be collected from recovered donors and employed to treat the infectious disease in question (2). In 2020, the worldwide spread of a previously unknown virus named SARS-CoV-2 caused the global COVID-19 pandemic. Experience from prior outbreaks with other coronaviruses (SARS-CoV-1) showed that convalescent sera contained neutralising antibodies (NAbs) against the virus and that their use was beneficial to the treated patients (3, 4). Therapy with antibody-laden blood of those who have recovered from SARS-CoV-2 infection is currently used and investigated worldwide (5–18).

Although convalescent plasma therapy has been considered generally beneficial due to multiple examples, both historical and recent (1, 19, 20), scientific medical community lacks definitive proof of its efficacy coming from carefully designed randomized clinical trials (2). When such trials were undertaken, they were unable to demonstrate a beneficial effect of plasma over placebo (21). The reasons lay mostly in the specific circumstances of its usage. Namely, the time frame of convalescent plasma usage is short. It is used only during epidemics caused by a new and insufficiently known pathogen, in a period when pathogen-specific therapy and vaccines are lacking. During this period, methods for plasma neutralisation potency determination are usually lacking or if they exist, they are neither standardized nor validated. This results in variability within the individual trials and renders comparison between different case studies, case series or trials difficult. Further, convalescent plasma is a complex, non-standardized medicine varying in NAb titre, as well as in the content of non-specific immunomodulators between units collected from different individuals. The inability to demonstrate convalescent plasma effectiveness might be linked to variation in the concentration of NAbs and the subsequent lack of standardized doses between patients (22, 23).

We describe here the Croatian approach to the establishing of prerequisites for the COVID-19 convalescent plasma (CCP) usage in a manner that enables comparison between different countries and studies. The approach includes several steps: (i) development of a reproducible wild-type SARS-CoV-2 neutralisation potency assay; (ii) establishment and continuous usage of an anti-SARS-CoV-2 in-house standard; (iii) finding the best fitting correlation function between the results of commercial assays used by Croatian transfusion centres and the results of neutralisation assay, thus enabling that the neutralisation potencies of plasma units can be expressed in the same way and in the same units in the whole country; and (iv) finally, and most importantly, recalculation of all neutralisation potencies of plasma units used in Croatia in relation to the first WHO international standard once it was established and available to the scientific community, by calibrating our own in-house standard (24).

## Materials and Methods

### Cell Media, Buffers and Solutions

Minimum Essential Medium (MEM; GIBCO, Thermo Fisher Scientific, MA USA) supplemented with penicillin (100 IU mL^-1^), streptomycin (100 µg mL^-1^), and L-glutamine (2 mM) (all from Capricorn Scientific, Germany) was used. Foetal bovine serum (PAN-Biotech, Germany) was inactivated at 56°C for 60 minutes prior the use. For the Vero E6 cell propagation and maintenance medium with 10% FBS was used. In SARS-CoV-2 titration (CCID_50_ assay) and SARS-CoV-2 neutralisation (ED_50_) assays medium was supplemented with 2.5% FBS.

### Cell Culture

Vero E6 cells, an epithelial cell line from the kidney of an African green monkey (*Ceropithecus aethiops*), were acquired from the American Type Culture Collection (ATCC – CRL 1587). The working cell bank was prepared, aliquoted and stored in liquid nitrogen. After thawing, cells were cultivated in cell cultivation flasks in the medium with 10% FBS in a 5% CO_2_ environment at 37°C, and maintained by subcultivations every 3-4 days, according to ATCC instructions.

### SARS-CoV-2 Working Stock

SARS-CoV-2 isolate was derived from the PCR positive oro- and nasopharyngeal swab designated 297/20 Zagreb taken from a patient in Zagreb, Croatia. It was propagated four times in Vero E6 cells. The virus was subcultivated one more time at m.o.i. 0.001 in Vero E6 maintained in MEM supplemented with 10% FBS, penicillin, streptomycin, and L-glutamine in a 5% CO_2_ environment at 37°C, to obtain SARS-CoV-2 working stock. Two days post infection virus-infected Vero E6 cell culture supernatant was centrifuged for 10 minutes at 1500×*g*, formulated in 20% FBS, aliquoted and stored at -75°C. It has been used throughout as a challenge virus in neutralisation assay, and as a reference in a virus microtitration assay.

### Next Generation Sequencing (NGS)

RNA vas isolated from 400 µL of virus-infected Vero E6 cell culture supernatants using Quick-RNA Viral kit (Zymo Research, USA). Reverse transcription and PCR amplification were performed following nCoV-2019 sequencing protocol for Illumina V.3 (available at https://www.protocols.io/), using LunaScript RT SuperMix kit (New England Biolabs, Germany) and Q5 Hot-Start High-Fidelity DNA polymerase (New England Biolabs) with primers from ARTIC nCoV-2019 V3 Panel (Integrated DNA Technologies, USA). Libraries were prepared with NEBNext Ultra II FS DNA Library prep kit (New England Biolabs), their quality was checked on 2100 Bioanalyser (Agilent, USA) using High Sensitivity DNA Kit (Agilent). Libraries were pooled and sequenced on Illumina MiniSeq instrument (Illumina, USA) using MiniSeq Mid Output Kit (2 × 150 paired-end reads; Illumina). Quality of raw reads was assessed with FastQC v0.11.8 and subjected to trimming, adapter removal and removal of short reads using BBDuk within BBTools package. Paired-end reads were aligned to hCoV-19/Wuhan/WIV04/2019 (GISAID accession ID EPI_ISL_402124) using Bowtie2 v2.4.2 (25). Geneious Prime^®^ 2019.2.3 software was used for construction of consensus sequences. The epidemiological lineage was attributed by the GISAID database, software version v.3.1.7 2021-07-09.

### Neutralisation Assay

Infective virus-neutralisation assay followed the general principles already described for other viruses (26, 27), but was adapted specifically to the SARS-CoV-2, as follows. SARS-CoV-2 neutralisation assay was performed in 96-well tissue culture microplates (TPP, Switzerland). Octaplicates of two-fold serial dilutions of the patient’s sera or the plasma donor’s serum (50 μL) were preincubated with approximately 20 CCID_50_/50 μL per well of SARS-CoV-2 working stock at 37°C and 5% CO_2_ for 90 minutes. Plasma of known neutralising capacity (anti-SARS-CoV-2 in-house standard) was used as a positive control (in duplicate). After adding 100 μL/well of Vero E6 cells (3×10^5^ mL^-1^), the plates were incubated at 37°C and 5% CO_2_. Wells with cell suspension without the virus served as cell growth control. After four days of incubation cell layers in all wells were inspected by an inverted optical microscope and wells with cytopathic effect (CPE) counted. The effective dose 50 (ED_50_), the amount of undiluted serum that inhibits CPE in 50% of infected wells, was calculated according to Spearman-Kärber method. Neutralisation titre (NT) was expressed as number of ED_50_ doses in 1 mL of plasma/serum. NTs of experimental samples in each assay were corrected for the deviation of anti-SARS-CoV-2 in-house standard from its nominal NT value. The quantity of SARS-CoV-2 working stock used as a challenge in each neutralisation assay was determined using 50% cell culture infective dose (CCID_50_) assay in 96-well format. Octaplicates of three-fold serial dilutions of virus suspension (100 μL) were mixed with Vero E6 cell suspension (3×10^5^ mL^-1^; 100 μL). After four days of incubation at 37°C and 5% CO_2_, wells with CPE were counted and CCID_50_ mL^-1^ calculated using Spearman-Kärber method. Anti-SARS-CoV-2 in-house standard was calibrated to the 1^st^ WHO International Standard for anti-SARS-CoV-2 (NIBSC, UK; https://www.nibsc.org/products/brm_product_catalogue/detail_page.aspx?catid=20/136), upon its availability, enabling expression of NT in IU mL^-1^.

### Commercial Serological Assays

Human IgG antibodies specific for SARS-CoV-2 spike (S) protein were determined by several commercial assays. Anti-SARS-CoV-2 ELISA (Euroimmun Medizinische Labordiagnostika AG, Germany) for determination of S-specific IgG uses S1 domain recombinantly produced in HEK 293 cells as a coating antigen. The VIDAS^®^ SARS-CoV-2 IgG (BioMerieux, France) is an automated ELFA assay, which uses recombinant receptor binding domain (RBD) of the S protein as the coating agent. The VIDAS^®^ SARS-COV-2 IgG test was performed on a VIDAS^®^ instrument. The LIAISON^®^ SARS-CoV-2 CLIA (DiaSorin Inc., USA) uses magnetic beads coated with recombinant S1 and S2 antigens and was performed on a Liaison XL analyser. Architect SARS-CoV-2 IgG II quant (Abbott, Ireland) is an automated assay using CMIA technique, in which receptor RBD of the S protein is used as a coating antigen. The assay was performed using Architect i2000SR instrument. Human IgG antibodies specific for nucleoprotein of the SARS-CoV-2 virus were measured by Abbott’s SARS-CoV-2 IgG assay, which uses recombinant nucleoprotein as a coating agent. We also measured human IgM and IgA antibodies specific for S protein, by BioMerieux’s VIDAS^®^ SARS-CoV-2 IgM and Euroimmun’s anti-SARS-CoV-2 ELISA (IgA) assays, respectively.

### Convalescent Plasma Samples

COVID-19 convalescent plasma (CCP) was obtained by apheresis using the Amicus (Fenwal, USA) and MCS+ (Haemonetics, USA) devices. Each donor had a documented history of laboratory-confirmed SARS-CoV-2 infection (positive RT-PCR test, positive SARS-CoV-2 antigen test, and/or SARS-CoV-2 specific antibodies). All plasma units were donated by recovered and healthy COVID-19 patients who had been asymptomatic for ≥ 28 days. If eligible according to standard blood donor criteria, donors were enrolled in a plasmapheresis program. Sera from eligible plasma donors were analysed by virus neutralisation assay for quantification of SARS-CoV-2 NAbs. Sera were analysed in native form and after inactivation by heating at 56°C for an hour. Collected plasma was used for therapy from donors having NT in heat-inactivated sample above 700 ED_50_ mL^-1^ (which equals 35 IU mL^-1^).

### CCP-Treated Patients

Demonstrated here are the NAb changes during the CCP therapy in the blood of three representative immunocompromised COVID-19 patients, who lacked their own antibodies. The first patient was diagnosed with nasopharyngeal diffuse large C-cell lymphoma and treated with R-CHOP/R-DHAP chemotherapy protocols (containing rituximab, cyclophosphamide, doxorubicin, vincristine and prednisone/rituximab, dexamethasone, cytarabine and cisplatin), followed by autologous stem cell transplantation, radiotherapy of Waldeyer’s ring, and finally maintenance therapy with the anti-CD-20 monoclonal antibody rituximab. At the start of CCP therapy a nasopharyngeal swab showed persistent RT-PCR positivity for SARS-CoV-2 for 45 days and he had a fever, respiratory insufficiency and lung infiltrates (28). The second patient had history of chronic lymphocytic leukaemia and was treated with rituximab and bendamustine. At the start of CCP therapy on the 57^th^ day of the disease, he had no anti-SARS-CoV-2 antibodies and was respiratory insufficient (dependent on low flow of oxygen therapy). The third patient had a history of follicular lymphoma treated with G-CHOP chemotherapy protocol (containing 2^nd^ generation anti-CD20 monoclonal antibody obinutuzumab, cyclophosphamide, doxorubicin, vincristine and prednisone), with the last cycle administered around the time of his infection with SARS-CoV-2 virus. He was diagnosed with COVID-19 pneumonia and treated with remdesivir and high flow oxygen therapy on several occasions. He lacked SARS-CoV-2 specific antibodies before the CCP transfusion, which was received on the 102^nd^ day of the disease.

### Data Analysis

NT values expressed as ED_50_ mL^-1^ or IU mL^-1^ were linearized by calculating logarithmic values and then used for statistical analysis. When average value from the set of *n* data was calculated, 95% confidence interval was provided as an indicator of measurement uncertainty. To assess a relationship of the antibody level with the donor disease severity and the time period from recovery to collection of CCP, Kruskal-Wallis test was performed. To assess a difference between heat inactivated and native sera a two tailed *t*-test for paired samples was used with *p* and *t* values, as well as degrees of freedom (DF) provided. Relationship between neutralisation assay results and results of other serological assays was assessed by Pearson’s correlation coefficient (*r*). MedCalc v20.011 was used for statistical analysis.

### Study Approval

Collection of COVID-19 convalescent plasma (CCP) was approved by the Croatian Ministry of Health (023-03/20-01/235; permission No. 534-04-3-2/2-20-11). The approval was based on the positive opinion of the Ethical Committee of Croatian Institute of Transfusion Medicine (003-06/20-04/02, opinion No.251-541-06/6-20-2). Immunocompromised COVID-19 patients were treated with COVID-19 plasma according to periodically updated FDA and EC guidelines (https://www.fda.gov/media/141477/download; https://ec.europa.eu/health/sites/default/files/blood_tissues_organs/docs/guidance_plasma_covid19_en.pdf). All COVID-19 convalescent plasma donors and all included patients (or their representatives) were informed about the study and gave written informed consent.

## Results

### Characterisation of SARS-CoV-2 Working Stock

The infectivity of SARS-CoV-2 working stock (the fifth (P5) subcultivation of 297/20 Zagreb SARS-CoV-2 isolate) was determined to be 6.83 ± 0.15 CCID_50_ mL^-1^ (*n*=8). Its complete genomic sequence was determined by NGS and compared to the sequence of the same virus isolate from the second subcultivation (P2), which is the closest one to the original clinical sample. Obtained genome coverage was 99.75% for both P2 and P5 samples. Sequences were submitted to GISAID database, accession IDs are EPI_ISL_3013040 and EPI_ISL_3013041 for P2 and P5, respectively. The virus belongs to the lineage B.1.1.1. Their consensus sequences were identical, except for nucleotides 4402 (ORF1ab gene) and 23607 (S gene). The difference stems from P5 ambiguity at both positions (C4402Y and G23607R in reference to P2; **Figure S1**). Both C and T at the position 4402 lead to the same amino acid in nsp3. The nucleotide 23607 is located in the S gene. Its ambiguity causes an amino acid substitution in a portion of the virus population (**Figure S1**). This amino acid is located within S1 protein subunit, but outside the RBD. In comparison to S protein of hCoV-19/Wuhan/WIV04/2019 [the sequence that represents the consensus of early submissions of SARS-CoV-2 sequences (29)] two differences were observed: G23607R (present only in P5) and A23402G (present in both P2 and P5). The latter difference is a well-known D614G mutation which occurred in early February 2020 (30) and is characteristic for nearly all strains detected since late spring 2020. The list of all nucleotide and amino acid differences between P5 and hCoV-19/Wuhan/Wiv04/2019 is presented in the **Table S1**.

Overall, we concluded that the virus has not substantially changed on its way from the clinical sample to the SARS-CoV-2 working stock, and can be considered as having the wild-type phenotype.

### Neutralisation Assay Development and Characterisation

Although there were plenty of different assays available on the market, none were sufficiently characterised to provide the information on the antibody quality and functionality. We focused on the establishment of stable and reproducible wild-type SARS-CoV-2 neutralisation assay, as the most relevant assay for estimation of the ability of antibodies to neutralise the virus infectivity. To gain reproducible and reliable results, we established laboratory working banks of Vero E6 cells, SARS-CoV-2 and anti-SARS-CoV-2 antibodies, aliquoted and stored in liquid nitrogen, at -60°C or below, and at -16°C or below, respectively.

**Figure 1:**
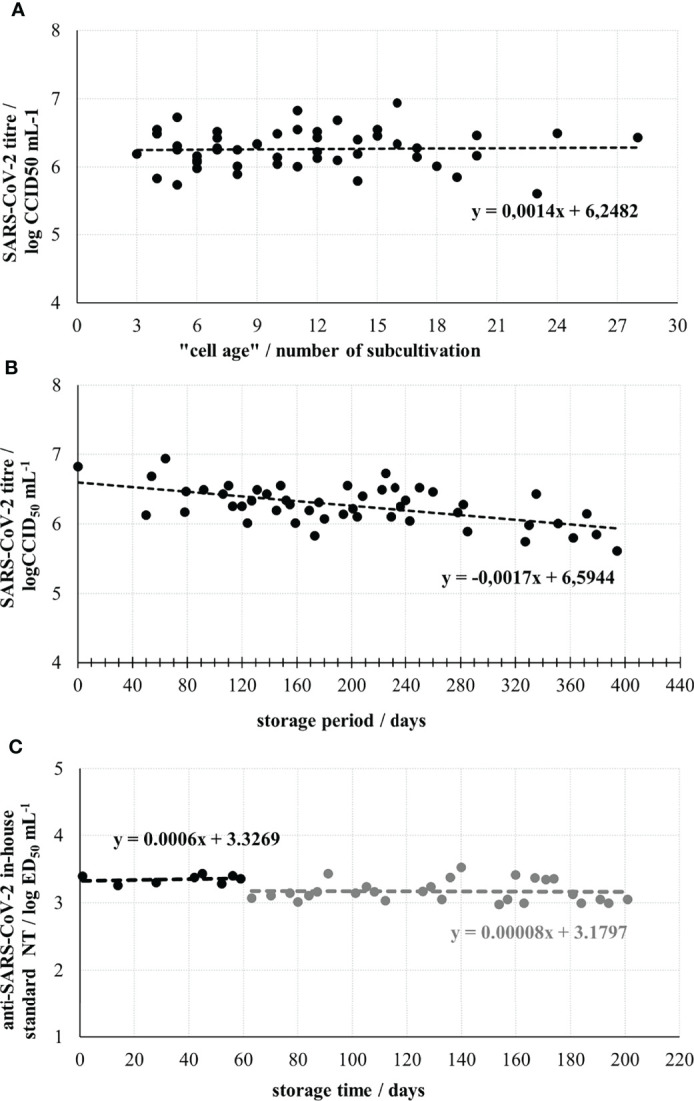
Optimization and control parameters of the neutralisation assay performance. **(A)** SARS-CoV-2 working stock titre determined in assay runs using Vero E6 cells from different subcultivations. **(B)** SARS-CoV-2 working stock stability during the storage at -60°C or below. **(C)** Stability of anti-SARS-CoV-2 in-house standard during 6-month storage. The break in line indicates the start of two times lower dilution of SARS-CoV-2 working stock usage as a challenge virus, due to the drop of its infectivity.

After thawing, Vero E6 cells were used for the assay from the 3^rd^ up to the 28^th^ subcultivation. The number of subcultivations did not significantly impact the titre of SARS-CoV-2 working stock, which was quantified in each assay run (**Figure 1A**). Linear function describing dependence of virus titre and “cell age” expressed as a number of subcultivations indicates a slight, negligible rise in titre value if cells for the assays were from 3^rd^ or 28^th^ subcultivation. In contrast, the titre of SARS-CoV-2 working stock has been continuously slowly dropping down with the increase of the storage period. Within the period of 9.3 months of storage at -60°C and below, the titre decreased from nominal 6.83 log CCID_50_ mL^-1^ to 6.21 log CCID_50_ mL^-1^, resulting in overall drop of approx. 0.61 log CCID_50_ mL^-1^, or 0.09 log CCID_50_ mL^-1^ per month (**Figure 1B**). This decrease was considered when preparing a working dilution of a challenge virus for the assay. Knowing that the results of the neutralisation assay inversely correlate with the titre of the challenge virus (26), we targeted 20 CCID_50_ per well (400 CCID_50_ mL^-1^), the estimated lowest amount ensuring 100% infectivity in all wells, which at the same time enables highest sensitivity of the neutralisation assay.

Anti-SARS-CoV-2 in-house standard was prepared by selecting one of the convalescent plasma samples, having a medium high NT in the preliminary assays. It was heat-inactivated, aliquoted and stored. Its nominal titre was determined to be 3.50 ± 0.04 log ED_50_ mL^-1^ (*n*=14). The NAb titre was stable throughout the 6-month period of its usage (**Figure 1C**). When the First WHO International Standard for anti-SARS-CoV-2 became available (NIBSC, UK, beginning of 2021) having nominal NT of 1000 IU mL^-1^ (4.32 ± 0.11 log ED_50_ mL^-1^ (*n*=11) in our assay), we calibrated our in-house standard to it. Several independently performed simultaneous analyses of both standards indicated that WHO International Standard, with 1000 IU mL^-1^ had 6.6 times higher neutralising activity than our in-house standard, to which 152 IU mL^-1^ was assigned accordingly. This enabled recalculation of all previously collected results and their expression in both ED_50_ mL^-1^ and IU mL^-1^, the units of the WHO International Standard.

### The Role of Complement in SARS-CoV-2 Neutralisation

**Figure 2:**
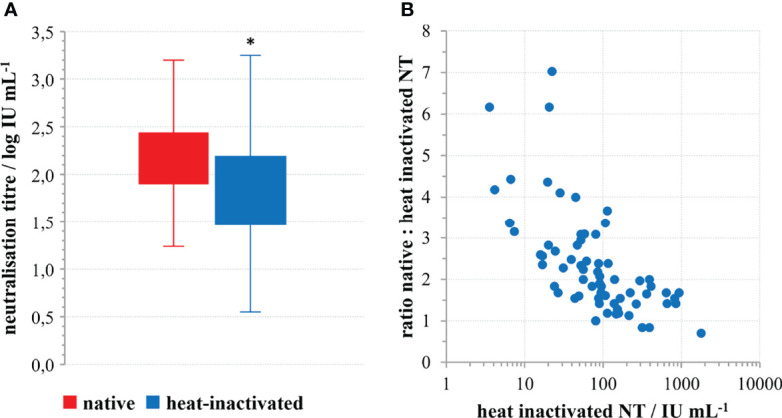
The role of complement in SARS-CoV-2 neutralisation. **(A)** Decrease of SARS-CoV-2 neutralisation power of convalescent sera upon heat inactivation; *p<0.0001 (*n*=69) according to the two-tailed *t*-test for paired samples; (*t*=12.809; DF=68), **(B)** Factor of neutralisation titre decrease in relation to the quantity of neutralising antibodies in the convalescents’ sera.

Analysis of 69 convalescent sera samples in native and heat inactivated form revealed 2.37 ± 0.30 (average ± 95% CI) times higher neutralisation potency of native samples (**Figure 2A**). The difference was statistically significant (*p*<0.0001; *t*=12.809; DF=68), indicating complement activation properties of convalescents’ SARS-CoV-2-specific antibodies. Moreover, the factor of neutralisation potency decrease upon complement inactivation was highest in samples with low neutralisation potency in comparison to the high titre sera (**Figure 2B**). This difference could be explained by the properties of the assay itself, since low titre samples are analysed in low dilutions in the assay, while high titre samples had to be highly diluted for analysis. In such situation, complement components and factors are also highly diluted, and their effect cannot be expressed equally as in low titre samples, analysed in low dilutions. So, it would be correctly to estimate complement activation properties of NAbs from the data obtained for low titre sera only (*n*=52), showing that neutralisation potency of specific antibodies is increased 2.67 ± 0.30 (average ± 95% CI) times, probably through classical activation of human complement. No complement activity against the SARS-CoV-2 was detected in the native human sera collected from people who were not infected or vaccinated.

### Neutralisation Potency of COVID-19 Convalescents’ Sera

Learning that the complement is activated by virus-specific antibodies and that it increases neutralisation potency, differently in high- and low-titre sera due to assay inherent properties, only heat-inactivated sera were tested to assess SARS-CoV-2 specific humoral immunity. We analysed in total 124 sera samples of COVID-19 convalescents by SARS-CoV-2 neutralisation assay who had a documented COVID-19 history and who successfully recovered. The time frame of plasma donation ranged between 1 to 7 months after recovery from the illness. Only four donors had unmeasurable quantities of NAbs in their blood, all four reported only mild symptoms and all four donated plasma within 3 months from the illness. The range of neutralisation potencies in positive sera was from 73 to 59452 ED_50_ mL^-1^ (1.87 to 4.77 log ED_50_ mL^-1^), which equals 4 to 2869 IU mL^-1^ (0.55 to 3.46 log IU mL^-1^). However, the median value was 1631 ED_50_ mL^-1^ (3.21 log ED_50_ mL^-1^) or 79 IU mL^-1^ (1.90 log IU mL^-1^).

**Figure 3:**
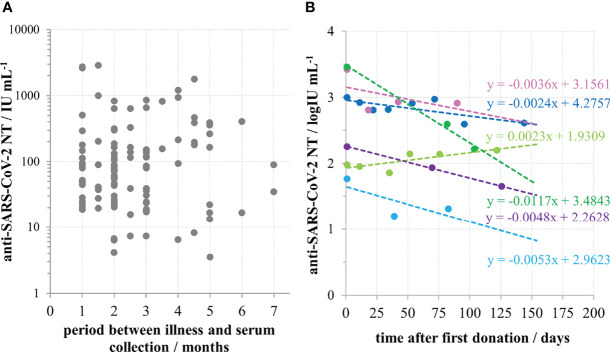
Persistence of anti-SARS-CoV-2 neutralizing antibodies in convalescent sera. **(A)** Neutralisation titre in convalescent donors’ sera (*n*=113) in relation to the time period between the illness and serum collection. **(B)** Longitudinal monitoring of neutralisation titre in sera of six convalescents.

**Figure 4:**
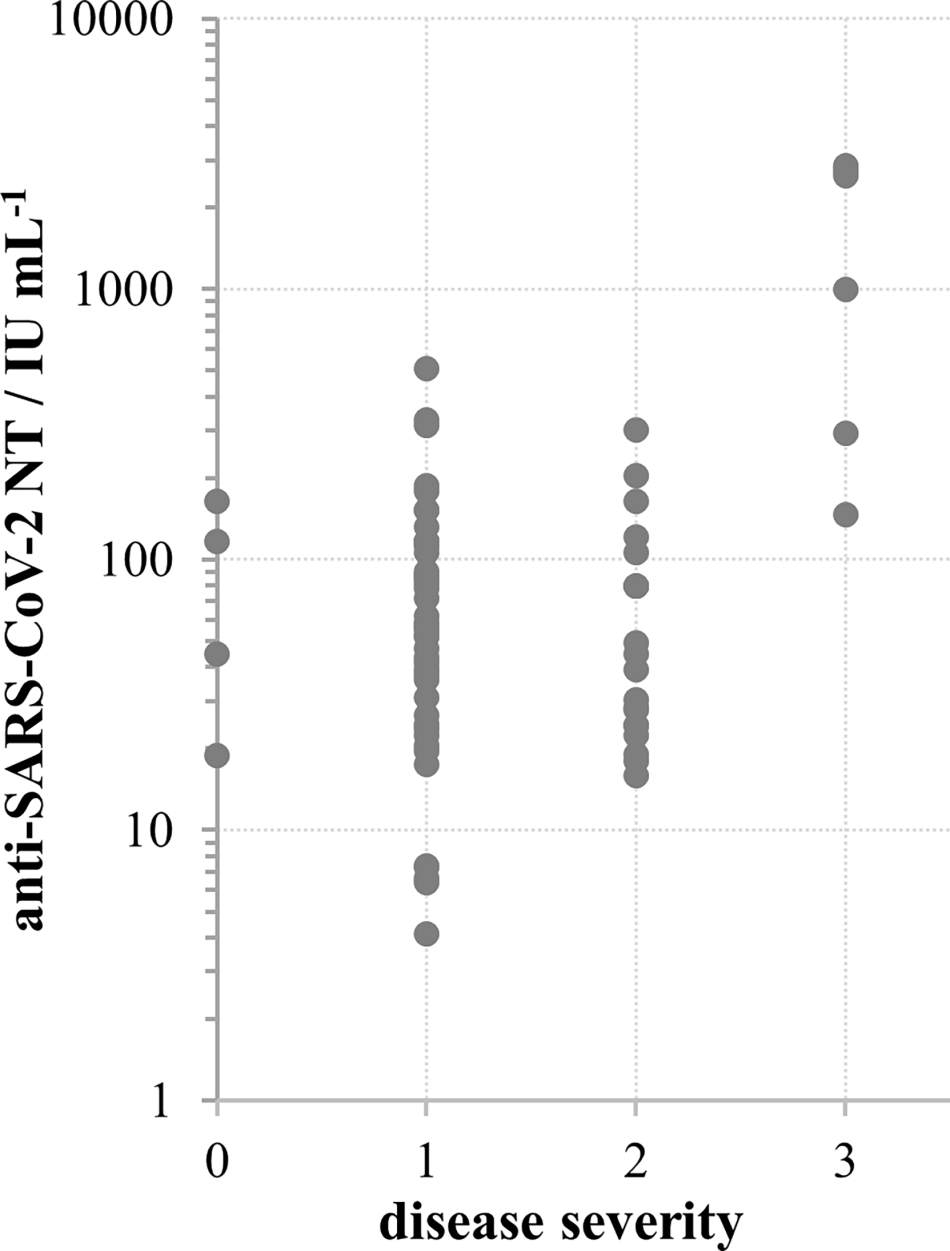
SARS-CoV-2 neutralisation titre in convalescent donors’ sera in relation to disease severity according to scale: 0 – no symptoms, 1 – mild, 2 – moderate, 3 – severe; *p < 0.002 in comparison to other groups, calculated by Kruskal-Wallis test.

For 113 samples we had complete data on the disease severity and the time of onset of their illness and used them to analyse the impact of these two factors on the NT level. NTs were highest in the group of sera taken within the first two months after the illness onset, then slowly decreased. However, measurable, even high levels of NTs were determined in samples taken 4-7 months after the illness onset (**Figure 3A**). If longer periods passed and antibodies were still high, it was highly probable that donors faced contact with the virus again which boosted their response, while having no symptoms due to the pre-existing immunity. To avoid such situations that might mislead our conclusion, only samples taken within first three months (*n*=77) were considered in the analysis of the impact of disease severity on NT. Disease severity was classified as asymptomatic (0), mild (1), moderate (2) and severe (3), on the basis of the donor’s report and on medical documentation (**Table 1**). Donors that reported the most severe clinical picture also had the highest levels of NTs (**Figure 4**) and this increase was statistically significant (*p*<0.002). However, most of them were not found eligible for donation due to their comorbidities. Furthermore, if their recovery was not complete for months after the illness, they were not considered for donation. Consequently, NAb titres in collected CCP units were not higher than 600 IU mL^-1^.

**Table 1:** Categories of COVID-19 disease severity.

Category	Designation	Symptoms
ASYMPTOMATIC	0	positive PCR test, no symptoms
MILD	1	short-term fever up to 38.5°C, anosmia, ageusia, runny nose, cough
MODERATE	2	short-term fever over 38.5°C, accompanied with several or all of the following: headache, mialgia, general weakness, vertigo and anosmia, ageusia, runny nose, cough
SEVERE	3	prolonged, persistent fever over 38.5°C, accompanied with the most of symptoms 2, involving also pneumonia in some cases; patients that seeked medical help, but without the need for hosptalization

Population picture (**Figure 3A**) implied that high NAb titres in convalescents persist for several months, and that their decrease is slow. This was proven by the analysis of duration of antibodies in six individual convalescents, showing that their neutralising antibodies were indeed persistent (**Figure 3B**). Slopes of lines describing the relationship between NT and the time after the first donation were in the range from -0.0117 to 0.0023, with an average value of -0.0042 log IU mL^-1^ per day.

### Relationship Between Neutralisation Potency and Commercial Assays for SARS-CoV2-Specific Antibody Determination

**Figure 5:**
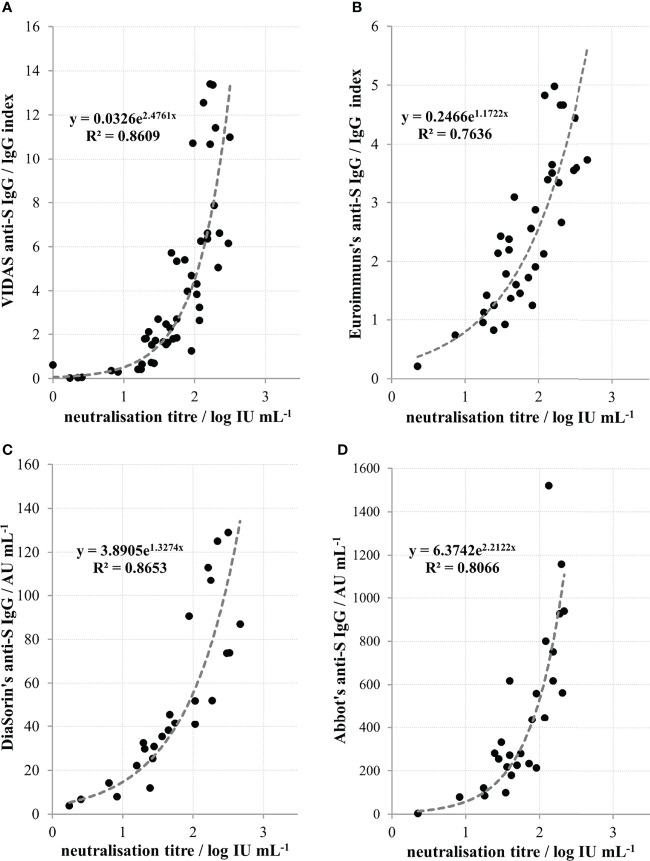
Relationship between neutralisation titres of convalescent sera and quantity of anti-spike protein (anti-S) IgGs determined by **(A)** Biomerieux’s VIDAS (*r*=0.93, *n*=49), **(B)** Euroimmun’s (*r*=0.87*; n*=36), **(C)** DiaSorin’s (*r*=0.93; *n*=25) and **(D)** Abbott’s (*r*=0.90; *n*=27) commercial assays. Coefficient *r* denotes Pearson’s coefficient of correlation from the *n* number of measurements.

COVID-19 convalescent plasma collection was initiated at the Croatian Institute of Transfusion Medicine in Zagreb. In parallel with the neutralisation potency determination of CCP plasma samples, several commercial assays compatible with instrumentation at this transfusion centre were also screened. We found high correlation (*r*=0.93; *n*=49) between the in-house neutralisation potency results of heat inactivated convalescent sera and the VIDAS SARS-CoV-2 IgG determining IgG specific for receptor binding domains of the SARS-CoV-2 S protein (**Figure 5A**). Due to the low throughput properties of the neutralisation potency assay, further characterisation of CCP samples was performed only by VIDAS SARS-CoV-2 IgG, and the results expressed in IgG index have been converted to NTs expressed in log IU mL^-1^ according to the inverse formula: y = 0.3477ln(x) + 1.4259, where x is IgG index. After the international standard became available, Biomerieux calibrated VIDAS SARS-CoV-2 IgG accordingly to it and their previous cut off value of 1.0 was replaced with 20 IU mL^-1^. Using the above formula describing the relationship between the NT and the SARS-CoV-2 IgG VIDAS assay results, we calculated similarly that the IgG index value of 1.0 equals 26.7 IU mL^-1^. This confirms that the correlation between VIDAS SARS-CoV-2 IgG and NT in COVID-19 convalescent sera is strong and that the transformation of ED_50_ mL^-1^ results to IU mL^-1^ is accurate.

NTs also significantly (*p*<0.0001) correlated to the results of several other commercially available serological assays that are used in other Croatian transfusion centres. A high correlation was found between NT of heat-inactivated convalescent sera and the results of Euroimmun’s anti-SARS-CoV-2 IgG (**Figure 5B**), DiaSorin’s anti-SARS-CoV-2 IgG (**Figure 5C**) and Abbott’s anti-SARS-CoV-2 IgG (**Figure 5D**), with Pearson’s coefficients of correlation being 0.87 (*n*=36), 0.93 (*n*=25) and 0.90 (*n*=27), respectively.

Results of commercial assay measuring IgG directed to the nucleoprotein of SARS-CoV-2 virus as well as of those measuring antibodies of IgM or IgA class specific for S protein did not correlate with the neutralisation potency results (data not shown).

### Convalescent Plasma Usage for COVID-19 Therapy

**Figure 6:**
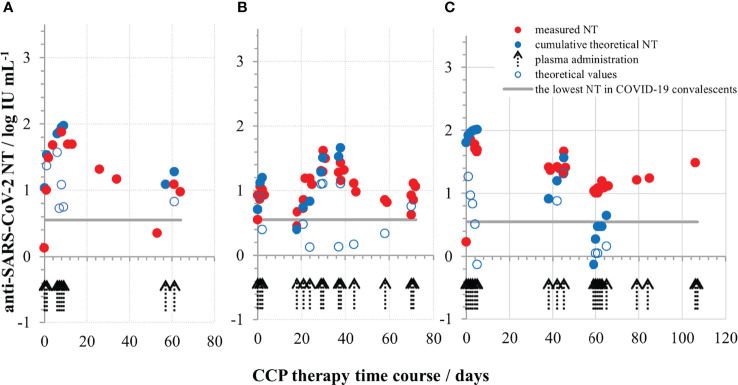
Neutralising SARS-CoV-2 antibody titres (in IU mL^-1^) during COVID-19 convalescent plasma therapy in the first **(A)**, second **(B)** and the third **(C)** representative COVID-19 patient, all three completely lacking their own immunoglobulins due to the underlying haematological malignancy and its treatment. The quantities of measured NAbs were above the lowest NT detected in successfully recovered persons.

In Croatia, the collection of convalescent plasma started in July 2020 and its use for COVID-19 therapy started in December 2020. A regularly updated document (last on 23/06/2020) “An EU programme of COVID-19 convalescent plasma collection and transfusion - Guidance on collection, testing, processing, storage, distribution and monitored use” was used as the main guidance document (https://www.isbtweb.org/fileadmin/user_upload/guidance_plasma_covid19_en.pdf). This most recent version still does not provide a cut-off value of NAb titre for the release of CCP for transfusion, due to lack of robust scientific evidence. When deciding which plasma units to provide to the hospitals the following assumptions were considered: (1) the lowest NT measured in sera of COVID-19 convalescents was around 4 IU mL^-1^, implying that such quantities might also be beneficial; (2) if the volume of one plasma unit is 200 mL and the average volume of human blood is 5-6 L, 25–30x dilution of NAbs is expected during transfusion; (3) the recruitment of convalescents for plasma donations was slow; (4) the median level of NT in convalescent plasma is closer to the lowest then to the highest measured value among the convalescents; (5) plasma units have to be given only to ABO compatible patients, which together with (3) and (4) would contribute to the insufficiency of units for the high needs during epidemics. The final decision was to consider plasma units with titres of 35 IU mL^-1^ and greater as suitable for transfusion, with a recommendation to provide the compatible plasma with the highest titre available at the moment and, if plasma units were of the lower titres, to provide several of them. COVID-19 convalescent plasma was given mostly to patients immunocompromised due to the different haematological malignancies or their treatment. Those lacking B-cell branch of immune system and immunoglobulins had no NAb before the CCP therapy and were excellent experimental systems to test the appropriateness of the above described recommendations. The increase of NAb levels in their serum samples after transfusion was successfully monitored, and measured quantities were in accordance to theoretical expectation, proving our decisions on plasma unit cut-off value and multiple units administration correct. Waves of NAbs were detected in the patients’ blood in quantities equal to the ones measured in some successfully recovered convalescents (three examples in **Figure 6**), proving that the here described approach indeed provided SARS-CoV-2 neutralising immunoglobulins to patients incapable of mounting these by themselves. Transfused NAbs were cleared from systemic circulation within the period of 3 weeks, after which plasma was given again if needed. NAbs induced improvement of clinical symptoms, either permanently or just transiently (28). Results on the effectiveness of COVID-19 convalescent plasma treatment of COVID-19 patients with underlying haematological malignancies will be reported separately.

## Discussion

Convalescent plasma (CP) usage to treat infectious diseases is limited to short periods of epidemics, when other treatment options are not available. These are the only times when clinical studies on CP application can be conducted, but in circumstances of inadequate characterisation of plasma units used for the treatment. In regular times drug investigated in clinical trials should be characterised by fully validated methods. In epidemics the methods are just being developed, with no calibration to international standards (because none exists) so CP is characterised in different, incomparable ways and sometimes used without any antibody characterisation. All this aggravates the assessment of clinical trial results (15). Currently, there is a growing number of trials on CCP usage for the treatment of COVID-19 hospitalized immunocompetent patients that have been completed or the interim results have been published and reviewed (31, 15). Most of them demonstrated no effectiveness of CCP over placebo, which is in line with the general principles of passive antibody therapy, based on a century long experience (3). Namely, antibodies have been considered more effective in prevention of infection diseases than in their treatment. If used for the treatment, they are more effective if provided before the onset of symptoms. Also, it has already been known that high antibody concentrations are required. Thus, the lack of evidence that CCP is beneficial in the treatment of COVID-19 severe patients should not come as a surprise, since the antibodies from a plasma unit (200 mL) are being diluted 25-30 times considering the full volume of human blood (cca 8% of human body weight). In addition, evidence is growing (including our work) that the quantity of SARS-CoV-2 specific antibodies is correlated with the severity of disease, highest in the most severe cases (32), implying that a small additional infusion cannot provide significant benefit. However, there are trials reporting beneficial outcomes of CCP usage for COVID-19 treatment (17, 33, 34), particularly those attributed to the high-titre CCP usage early in the course of the illness (13, 15, 16, 23). Currently collected data on the CCP treatment of COVID-19 in patients with innate or acquired immunosuppression have been promising and warrant further investigation (14, 18). It would be highly important to ensure that the plethora of available clinical data is comparable between different studies to correctly assess available results and make reliable conclusions for the future. This is currently not possible. For example, Li et al. reported the usage of CCP with S-RBD-specific antibody titre of at least 1:640 (not specifying assay and producer), which correlated to neutralisation titre (also not specifying the details of the assay) to some extent (*r*=0.622) (9). They reported that a serum NT of 1:80 is approximately equivalent to a titre of 1:1280 for S-RBD-specific IgG. Agarwal et al. treated patients with CCP without determining the antibody titre, but assayed them in a stored plasma samples at the end of the trial. They reported that 2/3 of donated plasmas had NT higher than 1:20, without stating many details of the assay itself (8). They also stated that most of their donors had only mild symptoms, implying low levels of antibodies in the blood given for transfusion. Simonovich et al. reported that infused plasma had median titre of 1:3200 obtained in COVIDAR ELISA measuring IgG specific for RBD of S protein. They demonstrated a certain, though not a high degree of correlation between this assay and the neutralising activity in a pseudotyped particle system (*r*=0.52) (10). Joyner et al. characterised antibody content in the plasma by the VITROS anti-SARS-CoV-2 enzyme immunoassay providing results in signal-to-cut off ratios. They categorized plasma units to low, medium and high, and observed the beneficial effect in patients treated with high antibody units (23). Obviously, COVID-19 convalescent plasma was used globally, but characterised by different assays, so the results cannot be compared.

In Croatia we established the wild-type virus neutralisation assay as the most reliable indicator of protective humoral immunity and used it in the screening of convalescent plasma units. ED_50_ assay format using 8 replicates of each two-fold dilution was preferred over plaque reduction neutralization assay due to its better resolution. Here we provide a detailed demonstration on the establishment of a highly controlled SARS-CoV-2 neutralisation assay. For the reduction of assay variability, the banking system of the main biological components of the assay – virus, cells and in-house antibody standard was used and they have been constantly controlled **Figure 1**). Involvement of the in-house standard in each assay and its use for the correction of experimental samples results ensured the assay’s reproducibility. Calibration of the in-house standard to the international standard as soon as it became available (24), enabled recalculation of all gained results for transfused plasmas to the units of the international standard, therefore expressing the results from our country in an internationally comparable way.

We demonstrate here that NAb level in plasma of COVID-19 convalescents (not including hospitalized patients) was in the range from 4 to 2869 IU mL^-1^ (0.55 to 3.46 log IU mL^-1^). We observed significant positive correlation between SARS-CoV-2 NTs in convalescents and the disease severity (**Figure 4**), already noticed by others (32, 35, 36). We also observed a slow decline of NAbs in COVID-19 recovered persons (**Figure 3A**), the kinetics of which were calculated from the data of longitudinal monitoring of NT levels in six individual patients (**Figure 3B**). Neutralising antibodies declined on average 1.4-times during one-month period, or *cca* 3-times during 3 months, which is in line with the estimation of Crawford et al. of 4-fold decline from 1 to 4 months after the symptom onset (37). Complement activity in COVID-19 has so far been addressed mainly from the pathology-inducing aspect (38, 39). Kang et al. also investigated the role of antibodies directed to SARS-CoV-2 nucleoprotein (40), based on the pre-printed hypothesis of Gao et al. that complement hyperactivation is induced *via* N-protein, and its binding to mannan-binding lectin-associated serine protease 2 (41). We demonstrated here that complement activation in convalescent sera is probably initiated by SARS-CoV-2 antibodies (**Figure 2**), resulting in titres 2.7 times higher in native than in heat-inactivated sera. High correlation between NTs in convalescent sera and results of several widely used serological assays determining IgGs specific to S protein was clearly demonstrated (**Figure 5**) corroborating the results of Šimanek et al. (42), while no correlation was found with assays determining other antibody classes or different antibody specificities. The cut-off value for the release of convalescent plasma to therapy was set to 35 IU mL^-1^. Food and Drug Administration authorized the emergency use of high titre COVID-19 convalescent plasma for the treatment of hospitalized patients with COVID-19 early in the course of disease and those hospitalized with impaired humoral immunity (August 23, 2020; revision on March 09, 2021; https://www.fda.gov/media/141477/download). High titre COVID-19 plasma, according to the current version, is the one with anti-SARS-CoV-2 IgG ratio in Euroimmun’s ELISA ≥ 3.5 or Abbott’s SARS-CoV-2 IgG result ≥ 840 AU mL^-1^. These values correspond to 144 and 131 IU mL^-1^, respectively, according to here determined relationships between the results of these two assays and neutralisation titres (**Figures 5B, D**). We can indirectly conclude that the value of high titre plasma expressed in units of the First WHO International Standard for anti-SARS-CoV-2 would be approx. 140 IU mL^-1^. In comparison to FDA recommendations, our limit for plasma usage was four-fold lower. However, patients were always given several units, thus multiplying the quantity of NAbs received. In RECOVERY trial plasma donations with a sample to cut-off ratio of 6.0 or more on the Euroimmun IgG ELISA were used for the treatment (7). We could estimate from the function describing significantly correlating relationship between NTs and Euroimmun’s results (**Figure 5B**) that plasmas of 323 IU mL^-1^ or more were used, but this is only estimation. True determination should be performed by assessing international standard in the Euroimmun’s assay, used in this particular trial.

In conclusion, a high amount of currently incomparable data has been generated during the previous two years. We should put our efforts to validate all different assays used in different studies and calibrate them to the First WHO International Standard as to enable conclusions on quantities of antibodies providing or not providing benefit in the treatment of COVID-19 immunocompetent and particularly immunocompromised patients.

## Data Availability Statement

The datasets presented in this study can be found in online repositories. The name of the repository and accession numbers can be found below: Global Initiative on Sharing Avian Influenza Data (GISAID) database, https://www.gisaid.org/, EPI_ISL_3013040 and EPI_ISL_3013041. All users of GISAID databases are issued personal access credentials after having provided their identity and agreed to terms of use that govern the GISAID sharing mechanism.

## Ethics Statement

The studies involving human participants were reviewed and approved by Croatian Ministry of Health (023-03/20-01/235; permission No. 534-04-3-2/2-20-11). The approval was based on the positive opinion of the Ethical Committee of Croatian Institute of Transfusion Medicine (003-06/20-04/02, opinion No.251-541-06/6-20-2). Immunocompromised COVID-19 patients were treated with COVID-19 plasma according to periodically updated FDA and EC guidelines (https://www.fda.gov/media/141477/download; https://ec.europa.eu/health/sites/default/files/blood_tissues_organs/docs/guidance_plasma_covid19_en.pdf). All COVID-19 convalescent plasma donors and all included patients (or their representatives) were informed about the study and gave written informed consent. The patients/participants provided their written informed consent to participate in this study.

## Author Contributions

SR established and performed the SARS-CoV-2 neutralisation assay. AH organized the convalescent plasma collection and its characterisation by different commercial serological assays, and managed distribution of plasma units toward clinicians. TK, OĐR, TM, and KK determined the anti-S antibodies using different commercial serological assays. TV and IJ were responsible for legislation connected to the plasma collection and usage. DR, OJ, GD, and ES treated COVID-19 patients using CCP. ŽMŠ isolated the wild-type SARS-CoV-2 virus from the clinical sample and subcultivated it. IK and AM ensured the continuous availability of BSL3 resources. JIJ, DF, and AS performed the NGS analysis of SARS-CoV-2 virus used in this study. BH orchestrated the whole work, collected and analysed the data and wrote the manuscript. The whole team participated in its shaping. All authors contributed to the article and approved the submitted version.

## Funding

The research was funded by the Croatian Science Foundation (grant IP-CORONA-04-2053 to BH) and by the European Regional Development Fund, grant number KK.01.1.1.01.0006, “Strengthening the capacity of CerVirVac for research in virus immunology and vaccinology”.

## Conflict of Interest

The authors declare that the research was conducted in the absence of any commercial or financial relationships that could be construed as a potential conflict of interest.

## Publisher’s Note

All claims expressed in this article are solely those of the authors and do not necessarily represent those of their affiliated organizations, or those of the publisher, the editors and the reviewers. Any product that may be evaluated in this article, or claim that may be made by its manufacturer, is not guaranteed or endorsed by the publisher.
