# Supplementary material for: Is Better Standardization of Therapeutic Antibody Quality in Emerging Diseases Epidemics Possible?
Source: Front Immunol. 2022 Feb 22;13:816159. doi: 10.3389/fimmu.2022.816159 (PMC8902244; doi:10.3389/fimmu.2022.816159)
Supplement: Supplementary file 1 [file Image_1.pdf]

A) 4402

orf1ab gene

nt 4402 ↓

**P2**	CGA	GAA	ATG	CTC	GCA	CAT	GCA
**P5**	CGA	GAA	ATG	CTY	GCA	CAT	GCA

nsp3 protein

aa 561 ↓

**P2**	R	E	M	L	A	H	A
**P5**	R	E	M	L	A	H	A

B) 23607

S gene

nt 23607 ↓

**P2**	AAT	TCT	CCT	CGG	CGG	GCA	CGT
**P5**	AAT	TCT	CCT	CRG	CGG	GCA	CGT

spike protein

aa 682 ↓

**P2**	N	S	P	R	R	A	R
**P5**	N	S	P	R or Q	R	A	R

Figure S1. Nucleotide differences between P2 and P5 consensus sequences (at genomic positions (A) 4402 and (B) 23607) and corresponding segments of nsp3 and spike proteins. Nt, nucleotide; aa amino acid.
