# Supplementary material for: Is Better Standardization of Therapeutic Antibody Quality in Emerging Diseases Epidemics Possible?
Source: Front Immunol. 2022 Feb 22;13:816159. doi: 10.3389/fimmu.2022.816159 (PMC8902244; doi:10.3389/fimmu.2022.816159)
Supplement: Supplementary file 2 [file Table_1.pdf]

Table S1. Genomic and protein differences between hCoV-19/Wuhan/WIV04/2019 and P5 (GISAID acc. ID EPI_ISL_402124 and EPI_ISL_3013041, respectively). The first letter indicates nucleotide or amino acid identity in hCoV-19/Wuhan/WIV04/2019. For synonymous substitutions only the number of corresponding amino acid is shown.

genomic region	nucleotide difference	protein	amino acid difference
5'UTR	C241T	n.a.	n.a.
orf1ab	C3037T	nsp3	106
orf1ab	C4002T	nsp3	T428I
orf1ab	T4402Y	nsp3	561
orf1ab	G10097A	nsp5 (3C-like proteinase)	G15S
orf1ab	C13536T	nsp12 (RNA-dependent RNA polymerase)	32
orf1ab	C14409T	nsp12 (RNA-dependent RNA polymerase)	P323L
S gene	A23403G	spike	D614G
S gene	G23607R	spike	682, R682Q
S gene	C23731T	spike	723
NS8 gene	G28209T	NS8	E106stop*
N	GGG28881-28883AAC	nucleocapsid	RG203-204KR

5'UTR, 5' untranslated region
n.a., not applicable
*truncation of NS8 protein for 16 amino acids
